# Validation of the Korean Version of the Anticipatory and Consummatory Interpersonal Pleasure Scale in Non-help-seeking Individuals

**DOI:** 10.3389/fpsyg.2022.859234

**Published:** 2022-04-29

**Authors:** Eunhye Kim, Diane C. Gooding, Tae Young Lee

**Affiliations:** ^1^Department of Psychiatry, Yangsan Hospital, Pusan National University, Yangsan, South Korea; ^2^PATHS Laboratory, Department of Psychology, University of Wisconsin-Madison, Madison, WI, United States; ^3^Department of Psychiatry, University of Wisconsin-Madison, Madison, WI, United States; ^4^Research Institute for Convergence of Biomedical Science and Technology, Yangsan Hospital, Pusan National University, Yangsan, South Korea

**Keywords:** Anticipatory and Consummatory Interpersonal Pleasure Scale (ACIPS), social anhedonia, social reward, validation, interpersonal pleasure

## Abstract

The Anticipatory and Consummatory Interpersonal Pleasure Scale (ACIPS) is a psychometric instrument that has been used to indirectly measure social anhedonia in many cross-cultural contexts, such as in Western (US), European (French, Spanish), Eastern (Chinese), and Israeli samples. However, little is known about the psychometric properties of the ACIPS in Korean samples. The primary goal of this study was to validate the Korean version of the ACIPS among non-help-seeking individuals. The sample consisted of 307 adult individuals who had no current or prior psychiatric history. Participants were administered the ACIPS, along with the Behavioral Inhibition and Behavioral Activation Scales (BIS/BAS) and Beck Depression Inventory (BDI). We examined the association of the total ACIPS scores with the other measures. The ACIPS showed good internal consistency. We also explored the factor structure of the Korean translation of the ACIPS using principal component analysis with Promax rotation and Kaiser normalization. Factor analysis yielded a three-factor structure that accounted for 58.8% of the variance. The three-factor model included the following subdomains: interactions involving close relationships, casual interactions, and interactions involving family members. Total BAS and BIS scores were significantly associated with total ACIPS scores, while BDI scores were inversely associated with total ACIPS scores. The current research indicates that the Korean version of the ACIPS is a useful and valid scale. Future directions include using the Korean translation of the ACIPS to elucidate the varying degrees of hedonic capacity in psychiatric patients.

## Introduction

Schizophrenia is a severe and persisting mental illness characterized by a neurocognitive deficits, functional decline, and a constellation of positive and negative symptoms, with up to 60% of patients experiencing negative symptoms (Correll and Schooler, 2020). Negative symptoms include anhedonia, apathy, avolition, blunted or restricted affect, and attentional impairment (Andreasen, 1982; Bobes et al., 2010). Anhedonia, defined as the absence or diminution of the ability for pleasure, is one of the prominent symptoms of patients with major depressive disorder and schizophrenia (Andreasen, 1982; Leventhal et al., 2006; Winograd-Gurvich et al., 2006; Horan et al., 2008; Zhornitsky et al., 2012).

There is increasing evidence that social anhedonia, i.e., decreased interest in and/or reduced reward from social interactions, is distinct from other forms of anhedonia (Gooding and Pflum, 2014a,b, 2022). Social anhedonia is an indicator of heightened risk for the later development of a schizophrenia-spectrum disorder (Kwapil, 1998; Gooding et al., 2005, 2007). It appears across all phases of schizophrenia-spectrum disorders, including the prodromal phase (e.g., Jhung et al., 2016). Findings from patients (e.g., Blanchard et al., 2001; Ritsner et al., 2018), first-degree relatives of patients with schizophrenia (e.g., Docherty and Sponheim, 2014; Umesh et al., 2018), and psychometrically identified schizotypes (e.g., Gooding et al., 2005) provide compelling evidence that social anhedonia is a trait-related symptom for many individuals within the schizophrenia spectrum. Thus, evaluating social anhedonia can be crucial in terms of understanding contributory aspects underlying schizophrenia-spectrum disorder.

Social anhedonia is also observed among several other forms of psychopathology (Barkus and Badcock, 2019; Liang et al., 2020; Gooding and Pflum, 2022). Currently, there is strong evidence that social anhedonia is a state-related symptom in patients with depressive disorder, though there are increasing reports of social anhedonia in autism, eating disorders, post traumatic stress disorder (PTSD), and substance use disorders. While there are several self-report measures designed to assess anhedonia, to date there are two validated scales specifically designed to measure social anhedonia. The Revised Social Anhedonia Scale (RSAS; Eckbland et al., 1982) is a direct measure of social anhedonia, whereas the Anticipatory and Consummatory Interpersonal Pleasure Scale (ACIPS; Gooding and Pflum, 2014a,b) is an indirect measure. Scores on the RSAS and ACIPS are strongly and negatively correlated with each other, though the two measures tap distinct aspects of social anhedonia (Gooding and Pflum, 2014a,b; Gooding et al., 2015, 2017). Some characteristics of the ACIPS distinguish it from the RSAS. First, the ACIPS was developed to assess individual differences in social pleasure, while the RSAS was originally developed to detect proneness to develop psychosis. Second, the ACIPS contains more updated content, measuring individuals’ ability to experience pleasure from social and interpersonal interactions that occur either in person or remotely (e.g., while texting). Recently, bivariate biometric analyses on twin data revealed that the ACIPS captures unique heritable contributions to social/interpersonal pleasure, in addition to sharing genetic variance with other self-report measures of positive affect (Gooding et al., 2021).

The ACIPS has been used in many cross-cultural contexts, such as in Western (US), European (Spanish), Eastern (Chinese), and Middle East (Israeli) samples. The internal consistency of the ACIPS has been high across all these contexts, regardless of whether the ACIPS was administered in English, European Spanish, Mandarin Chinese, or Hebrew, with ordinal alpha ranging from 0.85 to 0.96 (Chan et al., 2016; Gooding et al., 2016, 2017; Ritsner et al., 2018).

A few studies of the psychometric properties of the ACIPS have been conducted in cross-cultural contexts. For example, one research group (Gooding et al., 2016) examined the factor structure and construct validity of the ACIPS in a non-clinical Spanish adult sample. Using parallel analysis and maximum factor extraction, their analysis revealed a three-factor solution: Intimate Social Relations, Social bonding in the Context of Media, and Casual Socializing. Chan et al. (2016) examined the factor structure of the ACIPS in their non-clinical Chinese adult sample while also relying upon parallel analysis. They found a four factor structure characterized by Friendship, Family and Intimacy-Related Relationships, General Social Interactions, and Casual Interactions/Conversations. Chaix et al. (2017) compared one-, two-, and three- factor models using estimate fit testing for the French translation of the ACIPS. The best fitting solution was a three-factor model identifying Intimate Social Relations, Group Social Interactions, and Social Bonding and Making Connections as the factors. Thus far, there have been three cross-cultural validations of the ACIPS and they have been consistent in indicating the overall robustness of the measure.

Until now, the ACIPS has not been administered to any Korean samples. Prior to using the ACIPS in studies of Korean patients with schizophrenia-spectrum disorders, it is imperative that investigators first validate a Korean translation of the scale in a non-clinical sample. Therefore, the goal of the present study was to validate the Korean translation of the ACIPS for use with Korean populations. Given the previous findings that the translated versions of the ACIPS, like the original English version, are characterized by high internal consistency, we predicted that the Korean translation of the ACIPS would also be characterized by high internal consistency. We also hypothesized that factor analysis of the Korean translation of the ACIPS would reveal at least three but no more than four factors which could account for at least 50% of the variance. As a further examination of construct validity and measure of fidelity with the original English version of the ACIPS, we also examined the relationship between the Korean translation of the ACIPS with other measures of approach (the BAS), withdrawal (BIS), and affect (the BDI). Based on prior research (Gooding and Pflum, 2014b; Gooding et al., 2016, 2021), we expected the Korean version of the ACIPS to be associated with a measure of approach and reward sensitivity (i.e., the BAS) and withdrawal (the BIS) and inversely associated with a measure of negative affect (the BDI). No other *a priori* predictions were made.

## Materials and Methods

### Participants

The sample consisted of 307 (108 male, 199 female) non-clinical participants who were recruited from an online survey platform in South Korea in August 2021. These participants had previously agreed to be notified about survey opportunities through email announcements. After enrolling in the online survey platform, participants who met study criteria were invited to take the questionnaires. All participants were aged 17 years and older; the mean age of the sample was 25.1 (± 1.01) years. Exclusion criteria included no current or prior psychiatric history. The study was conducted as an online survey, with all individuals providing their informed consent through button press after the study was explained to them. This study was approved by the ethics committees of the Pusan National University and the Yangsan Hospital Institutional Review Board.

### Measures

The investigation included the following questionnaires: the Anticipatory and Consummatory Interpersonal Pleasure Scale (ACIPS; Gooding and Pflum, 2014a,b), the Behavioral Inhibition and Behavioral Activation Scales (BIS/BAS; Carver and White, 1994), and the Beck Depression Inventory-II (BDI-II; Beck et al., 1996b).

The ACIPS is an indirect measure of social anhedonia that is suitable for use in patient groups and non-clinical samples as well as different age groups (i.e., children, adolescents, and adults). The adult version of the ACIPS (Gooding and Pflum, 2014a,b) is a 17-item self-report measure in which hedonic capacity for social and interpersonal engagement is rated on a Likert-like scale of 1 (“very false for me”) to 6 (“very true for me”). Lower scores are associated with higher levels of social anhedonia. Translation of the ACIPS from English to Korean was conducted according to the international guidelines for translation of psychological measures (Hambleton et al., 2005). Two bilingual academic psychologists independently translated the ACIPS from English to Korean. The translations were reconciled and subsequently back-translated by an independent translator (another bilingual academic psychologist) in English who had not previously seen the original English version of the questionnaire. The back translations were reviewed with the primary developer of the measure (DCG) to verify the conceptual equivalence between the English and Korean versions of the ACIPS.

The Korean version of the BIS/BAS was used to examine convergent validity (Carver and White, 1994). The BIS/BAS consisted of 24 items that were rated on a Likert-type scale from 1 (very true) to 4 (very false). The BAS consisted of three subscales: Reward Responsiveness, Drive, and Sensation Seeking. The ordinal alpha for the BIS was 0.84. The ordinal alpha values for the BAS were 0.70, 0.69, and 0.80 for the Reward Responsiveness, Fun-Seeking, and Drive subscales, respectively.

The BDI-II (Beck et al., 1996a) is a self-report questionnaire based on the symptoms described by the Diagnostic and Statistical Manual of Mental Disorders-IV. The BDI consists of 21 items, in which four response options range from 0 to 3. Higher scores reflect greater symptom severity. The BDI showed a high level of internal consistency. The ordinal alpha for the BDI was 0.95.

### Statistical Analysis

We conducted an exploratory factor analysis on the ACIPS items using principal component analysis with Promax rotation and Kaiser normalization. We examined the internal consistency for the ACIPS, BAS/BIS, and BDI by calculating the ordinal version of alpha coefficients (Zumbo et al., 2007) because the measures had Likert-type response scales. We evaluated the construct validity of the ACIPS by examining the association of the total ACIPS scores with the BAS, BIS, and BDI total scores. All *p*-values are two-tailed. We used Meng’s test (Meng et al., 1992) to compare the strength of the correlations between the self-report measures. We used SPSS version 27 to perform the Meng’s test. The remaining data analyses were performed using R v4.1.2 statistical package.

## Results

### Demographics

Table 1 provides the demographic data for the sample. Independent samples *t*-test revealed that the mean total ACIPS scores of male (70.04 ± 15.25) and female participants (68.10 + 15.12) did not differ significantly, *t* (305) = 1.91, *p* = 0.26. Although the ages of participants ranged from 17 to 35 years, we observed no correlation between age and total ACIPS score in our sample, *r* = −0.02, *p* = 0.70. Accordingly, there was no significant between-group difference when participants were classified according to age, *F*(2, 304) = 0.33, *p* = 0.72.

**TABLE 1:** Mean Anticipatory and Consummatory Interpersonal Pleasure Scale (ACIPS) scores by demographic group.

	Total sample (*n* = 307)	Mean	*SD*
**Sex**			
Male	108	70.04	15.25
Female	199	68.1	15.12
**Age**			
17∼19	14	71.73	15.78
20∼29	134	68.31	15.06
30∼35	159	68.91	15.28

*Scores on the adult version of the ACIPS (Gooding and Pflum, 2014a,b) can range from 17 to 102, with lower scores reflecting greater likelihood of social anhedonia.*

### Internal Consistency

The total ACIPS showed good internal consistency, with an ordinal coefficient of 0.94. As expected, the ACIPS items assessing anticipatory pleasure were significantly associated with the ACIPS items assessing consummatory pleasure (*r* = 0.56, *p* < 0.001).

### Factor Structure of the Korean Translation of the Anticipatory and Consummatory Interpersonal Pleasure Scale

**FIGURE 1:**
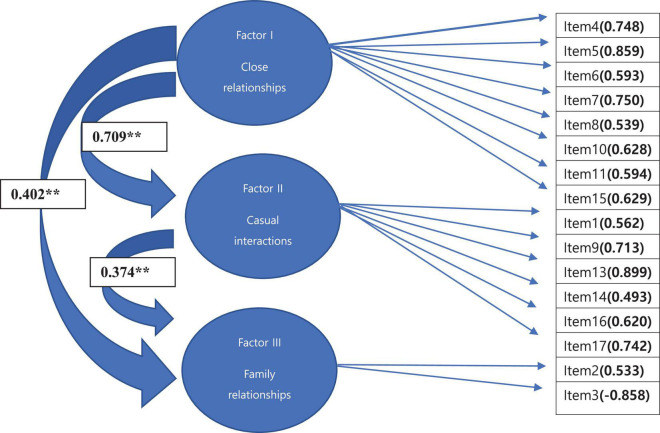
Three-factor model for the Korean translated ACIPS. **p < 0.01.

We explored the factor structure of the Korean translation of the ACIPS using factor analysis. Promax rotation of the ACIPS factor loadings using Kaiser normalization revealed a three-factor structure which together accounted for 58.81% of the variance. All three factors assessed both anticipatory and consummatory aspects of pleasure (Table 2). As indicated in both Table 2 and Figure 1, nine ACIPS items loaded onto Factor I (close relationships), which accounted for 47.13% of the variance. Factor II (casual interactions) contained six items, assessing social and interpersonal interactions of a more casual nature and accounted for 6.06% of the variance. Factor III (interactions concerning family members) contained only two ACIPS items but accounted for 5.61% of the variance in the total ACIPS scores. The first two factors appeared to be more highly correlated than the third factor (Table 3).

**TABLE 2:** Factor structure and loading of the ACIPS.

ACIPS item	Factor 1 Close relationships	Factor II Casual interactions	Factor III Family relationships
4.	**0.748**	-0.007	0.070
5.	**0.859**	-0.207	0.101
6.	**0.593**	0.124	0.090
7.	**0.750**	-0.015	0.079
8.	**0.539**	0.226	0.023
10.	**0.628**	-0.024	0.257
11.	**0.594**	0.262	-0.083
12.	**0.865**	-0.038	-0.079
15.	**0.629**	0.338	-0.234
1.	0.050	**0.562**	0.191
9.	-0.134	**0.713**	0.279
13.	-0.145	**0.899**	0.022
14.	0.300	**0.493**	-0.028
16.	0.199	**0.620**	-0.245
17.	0.027	**0.742**	0.019
2.	0.204	0.185	**0.533**
3.	-0.012	0.049	-**0.858**

*Rotated component matrix for the Korean version of the ACIPS. Extraction method = principal component analysis; rotation = Promax with Kaiser normalization; variance explained = 58.81%. The items that load on each factor are in boldface. ACIPS, Anticipatory and Consummatory Interpersonal Pleasure Scale; A, anticipatory pleasure item; C, consummatory pleasure item.*

**TABLE 3:** Correlations between the ACIPS factors.

	Factor I	Factor II	Factor III
Factor I	1.000		
Factor II	0.709**	1.000	
Factor III	0.402**	0.374**	1.000

*Associations between the Korean translation of the ACIPS factors. The three factors were empirically derived from factor analysis and are as follows: Factor I, close relationships; Factor II, casual interactions; Factor III, family relationships. All together, these factors accounted for 58.81% of the total variance. ACIPS, Anticipatory and Consummatory Interpersonal Pleasure Scale.*

**p < 0.01.

### Convergent and Discriminant Validity

Table 4 provides the descriptive statistics for the ACIPS, BAS/BIS, and BDI. To establish the convergent validity of the Korean translation of the ACIPS, we examined the associations between the ACIPS and a measure of approach-related affective behavior, namely, the BAS. Scores on the BAS were significantly correlated with ACIPS scores, *r* = 0.47, *p* < 0.01. The BIS of the BIS/BAS showed a small but significant association with total ACIPS scores, *r* = 0.03, *p* < 0.01. Further analyses revealed that the correlation between total ACIPS scores and total BAS scores was significantly stronger than the association between total ACIPS scores and total BIS scores, *Z* = 5.50, *p* < 0.001.

**TABLE 4:** Descriptive statistics for the psychometric measures (Mean ± *SD*).

		Total sample (*n* = 307)	Males (*n* = 108)	Females (*n* = 199)
Total ACIPS		68.78 (15.17)	70.04 (15.25)	68.10 (15.12)
	Anticipatory Consummatory	27.84 (5.49) 40.61 (9.27)	27.95 (5.98) 41.73 (9.49)	27.78 (5.23) 39.99 (9.13)
BAS		37.74 (6.77)	32.19 (7.08)	31.50 (6.59)
	Reward responsiveness Fun seeking Drive	5.63 (1.4) 9.05 (2.31) 10.02 (2.57)		
BIS		17.15 (3.24)	16.19 (3.44)	17.67 (3.01)
BDI-II		37.71 (10.93)	32.19 (7.08)	31.5 (6.59)

*Descriptive statistics are provided for the self-report measures used in the investigation: ACIPS, Anticipatory and Consummatory Interpersonal Pleasure Scale (Gooding and Pflum, 2014a,b); BAS, Behavioral Activation Scale (Carver and White, 1994); BIS, Behavioral Inhibition Scale (Carver and White, 1994); and BDI-II, Beck Depression Inventory-II (Beck et al., 1996a). Mean subscale scores for the ACIPS and the BAS are also provided. The group means (and standard deviations) are provided for the total sample as well as male and female participants separately.*

Total BDI scores were inversely associated with total ACIPS scores (*r* = −0.36, *p* < 0.01). The association between total ACIPS scores and BAS scores was stronger than the association between ACIPS scores and BDI scores, *Z* = 7.24, *p* < 0.001.

## Discussion

The main goal of this study was to validate the Korean translation of the ACIPS for use with Korean-speaking groups. This study provides the first evidence of cross-cultural validity of the ACIPS in a Korean-speaking context. Specifically, the results of the present study demonstrate that the adult version of the ACIPS could be successfully adapted from American English to Korean with semantic, linguistic, and contextual equivalence.

Our results revealed that the Korean translation of the adult version of the ACIPS had good internal consistency, as evidenced by the ordinal alpha coefficient. Results of exploratory factor analysis indicated a three-factor solution, namely, interaction in the context of close relationships, casual interactions, and family-related interactions. This factor structure is consistent with prior results of factor analyses conducted on different translations of the ACIPS. Overall, it appears that the ACIPS taps various types of social and interpersonal pleasure, including casual and intimate social interactions. It also appears that the factors include both anticipatory and consummatory items, rather than the ACIPS scale being subdivided into anticipatory and consummatory subdomains by factor analysis.

We did not observe any age effects on total ACIPS scores. However, there was not a large age range in the sample. Surprisingly, we did not observe any sex difference in the present sample. In prior reports based on adult samples, female respondents reported higher levels of social and interpersonal pleasure than males, regardless of age, or proportion of males in the sample. This pattern of sex difference was observed in American samples (Gooding et al., 2015), Spanish samples (Gooding et al., 2016), and Chinese samples (Chan et al., 2016). It is unclear whether the lack of sex differences in this Korean sample reflects a more “non-traditional” attitude toward gender roles in Korea (Choe, 2017) or whether it reflects a greater likelihood of a dampening of social pleasure amongst our female respondents.

In terms of total ACIPS mean scores, we observed lower mean scores in our Korean sample compared to other community-based adult samples, whether in the US (Bedwell et al., 2014; Gooding et al., 2015) or in China (Liang et al., 2020). We tried to identify reasons for the apparent differences in the mean total ACIPS scores. The differences in total mean scores cannot be attributed to differences in mode of survey administration (i.e., online versus in-person survey administration) because Gooding et al. (2015) used a similar mode of survey administration as the present study. One potential source of difference between the mean total ACIPS scores of the present sample (68.78 ± 15.17), the American sample of 305 adults (79.36 ± 13.40), and the Chinese sample of 74 adults (*M* = 74.1 ± 12.04) is the fact that our data collection occurred in the midst of the SARS-COVID-19 pandemic. This global epidemic may have had a dampening effect on the collective *joie de vivre* of an entire population. Indeed, it will be important to conduct further investigations of non-patient community-derived adults to determine whether these findings of relatively lowered total ACIPS scores reflect something about the sample itself or whether they reflect a cohort effect.

The Korean translation of the ACIPS displayed good construct validity. The total ACIPS scores were positively associated with total BAS scores, consistent with prior findings based on English versions of the measures (Gooding and Pflum, 2014a,b). The ACIPS was inversely associated with the BDI-II, consistent with a prior finding based on the Spanish version of ACIPS (Gooding et al., 2016). In the Spanish sample, participants who reported no or few depressive symptoms had significantly higher total ACIPS scores than those who reported moderate-to-severe depressive symptoms (Gooding et al., 2016). In terms of convergent validity, we expected that the ACIPS would be more closely related to measures of approach activation (i.e., the BAS) and inversely associated with scales that included direct measurement of anhedonia (i.e., the BDI) compared to measures of inhibition (i.e., the BIS).

### Limitations

There are a few limitations in the present study. First, we conducted this study using an online survey. We do not think this affected the results, given that online data collection can be an effective and valid means of data collection. However, it should be noted that all of the measures involved self-reported feelings, attitudes, and emotions. The self-report approach assumes that respondents can accurately reflect upon and rate their affective experiences. There are data that indicate a relationship between self-reported social and interpersonal pleasure using the ACIPS and electromyograhic response (Kadison et al., 2014). Although the participants were limited to those without current or prior psychiatric history, there was no other clinical characterization of the sample. In the future, it would be helpful to measure participants’ personality traits and clinical functioning to study the correlates of social hedonic capacity. Another potential limitation of the present study concerns our inclusion of some 17-year-olds in the sample. These individuals were included because they had matriculated from high school and therefore were considered “adult status.” We do not think this is a major limitation, given that only 3 of 307 (0.97%) participants fell into this category.

### Future Directions

The Korean translation of the ACIPS is now ready to be used in clinical populations, such as persons living with schizophrenia and groups of individuals with lived experience of severe mental illness. One of the advantages of the ACIPS is its temporal stability (*r* = 0.78; Gooding and Pflum, 2014b) over a period of approximately 5–8 weeks. This suggests that the ACIPS is suitable for use in evaluating patients before and after targeted intervention. Working memory impairments are posited to serve as a key contributor to social anhedonia in schizophrenia (Gooding, 2022; Gooding and Pflum, 2022). As such, any evidence-based interventions such as cognitive remediation (see Vita et al., 2021), which might be effective in improving the cognitive functioning of people with schizophrenia, may partly ameliorate some of their social anhedonia.

Similarly, impairments in social cognition may cause or exacerbate the social anhedonia that characterizes many individuals with serious and persistent mental illness, including, but not limited to, schizophrenia-spectrum disorders (Favrod et al., 2019; Gooding and Pflum, 2022). There is a paucity of research in this area. Interventions which target social cognition and include the ACIPS pre- and post-intervention along with behavioral measures of *in vivo* social interactions would be useful in this regard.

## Conclusion

Our results indicated that the ACIPS showed good construct validity in the Korean population. Future directions include using the Korean translation of the ACIPS to elucidate the varying degrees of hedonic capacity in patients with psychiatric disorders.

## Data Availability Statement

The original contributions presented in the study are included in the article/supplementary material, further inquiries can be directed to the corresponding author/s.

## Ethics Statement

The studies involving human participants were reviewed and approved by the Ethics Committees of the Pusan National University at Yangsan Hospital Institutional Review Board. The patients/participants provided their written informed consent to participate in this study.

## Author Contributions

TL and DG designed the research and critically revised the article for important intellectual content. DG conceptualized and developed the scale. EK and TL acquired the data. EK, TL, and DG analyzed and interpreted the data. EK drafted the first version of the manuscript. All authors approved the final version for publication, agreed to be accountable for all aspects of the work by ensuring that any questions related to its accuracy and integrity can be appropriately investigated and resolved.

## Conflict of Interest

The authors declare that the research was conducted in the absence of any commercial or financial relationships that could be construed as a potential conflict of interest.

## Publisher’s Note

All claims expressed in this article are solely those of the authors and do not necessarily represent those of their affiliated organizations, or those of the publisher, the editors and the reviewers. Any product that may be evaluated in this article, or claim that may be made by its manufacturer, is not guaranteed or endorsed by the publisher.
